# Supplementary material for: Nonequilibrium self-assembly dynamics of icosahedral viral capsids packaging genome or polyelectrolyte
Source: Nat Commun. 2018 Aug 6;9:3071. doi: 10.1038/s41467-018-05426-8 (PMC6078970; doi:10.1038/s41467-018-05426-8)
Supplement: Supplementary file 1 — Supplementary Information [file 41467_2018_5426_MOESM1_ESM.pdf]

# Supplementary Information: Nonequilibrium self-assembly dynamics of icosahedral viral capsids packaging genome or polyelectrolyte

Maelenn Chevreuil,[1,2] Didier Law-Hine,[1] Jingzhi Chen,[1] Stéphane Bressanelli,[2] Sophie Combet,[3] Doru Constantin,[1] Jéril Degrouard,[1] Johannes Möller,[4] Mehdi Zeghal[1] and Guillaume Tresset[1*]

[1]*Laboratoire de Physique des Solides, CNRS, Univ. Paris-Sud, Université Paris-Saclay, 91405 Orsay Cedex, France*

[2]*Institute for Integrative Biology of the Cell (I2BC), CEA, CNRS, Univ. Paris-Sud, Université Paris-Saclay, 91198 Gif-sur-Yvette Cedex, France*

[3]*Laboratoire Léon Brillouin (LLB), UMR 12 CEA-CNRS, Université Paris-Saclay, CEA-Saclay, F-91191 Gif-sur-Yvette Cedex, France*

[4]*European Synchrotron Radiation Facility (ESRF), 71 avenue des Martyrs, 38000 Grenoble, France*

[*]*e-mail:* guillaume.tresset@u-psud.fr

## Supplementary Figures

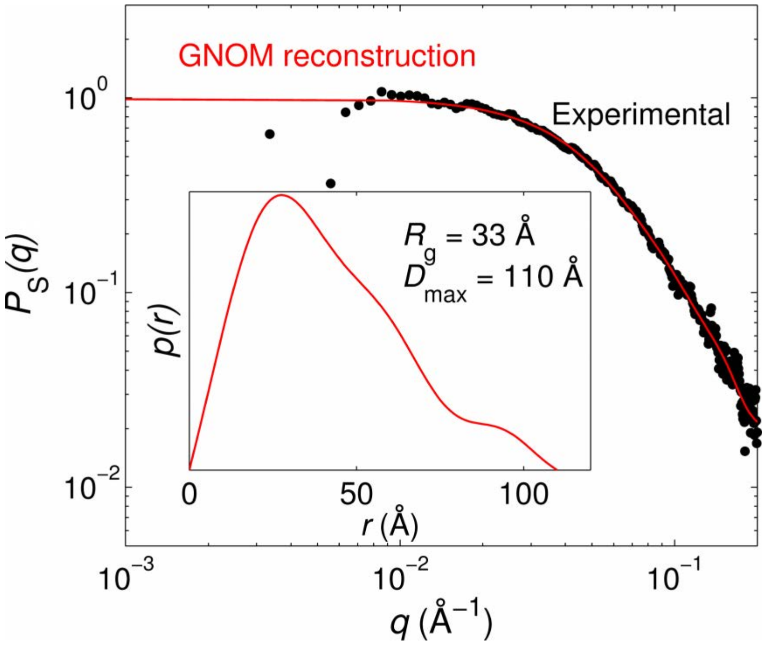


**Supplementary Fig. 1 | Form factor of the subunit.** CCMV dimers at a concentration of 25 μM were in a buffer solution at pH 7.5 and ionic strength 0.5 M. A reconstruction performed with the program GNOM[1] (red line) is compared to experimental data (black symbols). The inset shows the pair distribution function $p(r)$ computed by GNOM along with the calculated radius of gyration $R_g$ and the maximum dimension $D_{max}$.

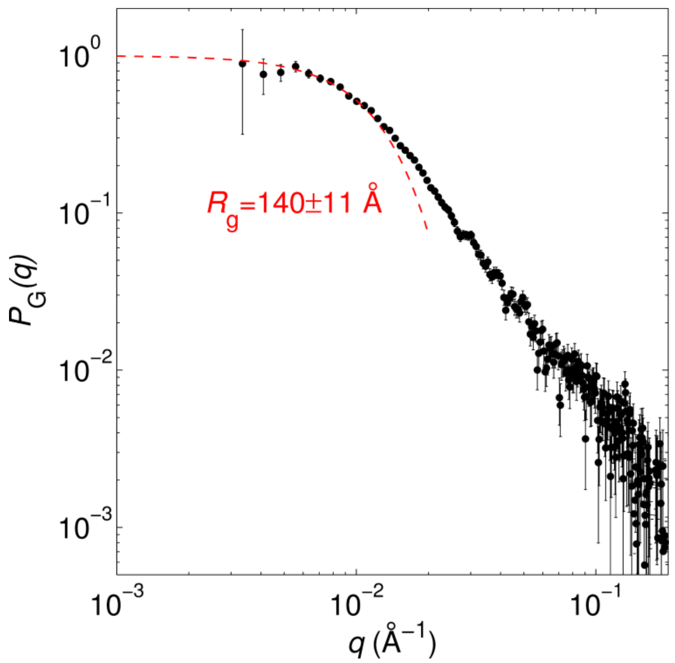


**Supplementary Fig. 2 | Form factor of the genome.** CCMV RNA at a concentration of 88 nM was in a buffer solution at pH 7.5 and ionic strength 0.1 M. The radius of gyration $R_g$ is estimated to be 140±11 (s.e.m.) Å by using Guinier approximation (red dashed line). Note that Guinier approximation is only valid for $qR_g < 1.3$, which means here $q < 9\times10^{-3}$ Å$^{-1}$. Error bars are defined as s.e.m. and were obtained by propagating the standard deviations of photon counts (see Methods).

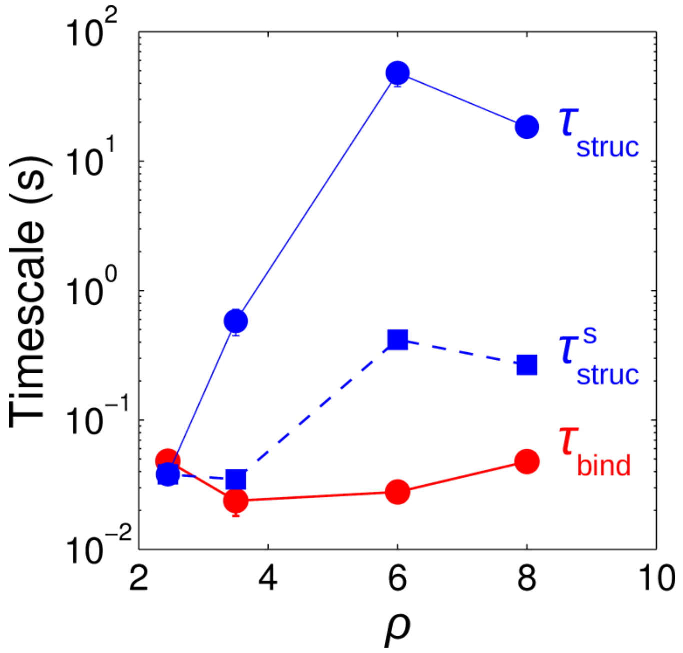


**Supplementary Fig. 3 | Characteristic timescales for the self-assembly dynamics of NPCs at various $\rho$.** Binding time $\tau_{\text{bind}}$, structural time $\tau_{\text{struc}}$ and shortest structural time $\tau^{\text{s}}_{\text{struc}}$ as a function of the mass ratio $\rho$ in a buffer solution at pH 7.5 with a final ionic strength of 0.1 M. $\tau_{\text{bind}}$ was obtained by fitting $\langle N \rangle_{\text{up}}$ with a single exponential decay function while $\tau_{\text{struc}}$ and $\tau^{\text{s}}_{\text{struc}}$ were obtained by fitting $R_{\text{g}}$ with a double exponential decay function. Error bars are defined as s.e.m. and were obtained by propagating the standard deviations of photon counts (see Methods).

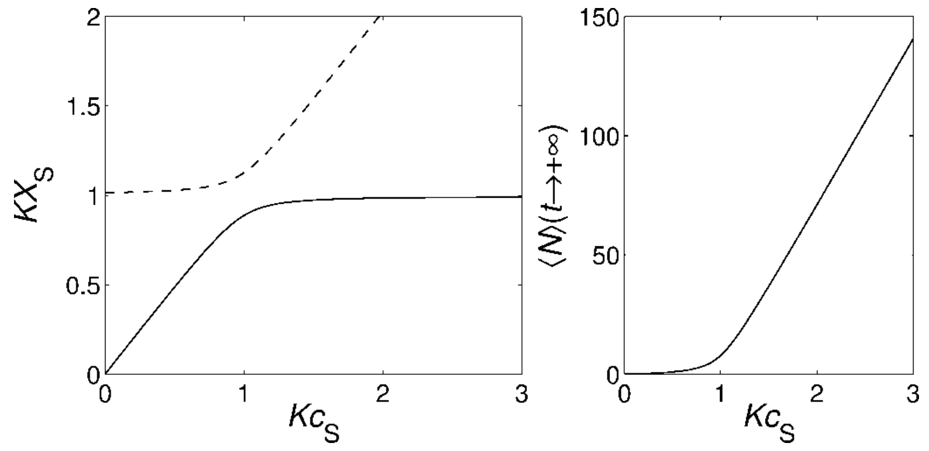


**Supplementary Fig. 4 | Critical concentration at the equilibrium.** Solution of Supplementary Equations 4 and 6 with $c_G = 0.2$ µM and $K^{-1} = 14$ µM, which are typical concentrations encountered in the experiments. The dashed line is an unstable branch.

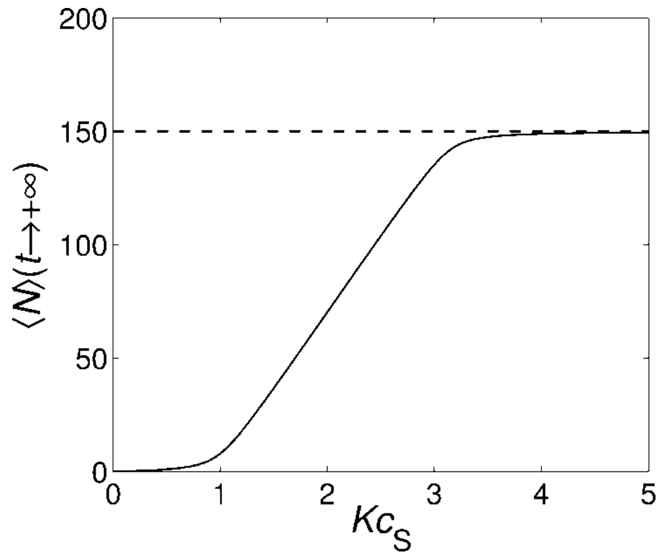


**Supplementary Fig. 5 | Critical concentration and saturation at the equilibrium.** Solution of Supplementary Equations 9 and 6 with $c_G = 0.2$ µM, $K^{-1} = 14$ µM and $N_{max} = 150$. This latter value corresponds to the charge neutralisation of the genome by the RNA-binding domain of the bound subunits.

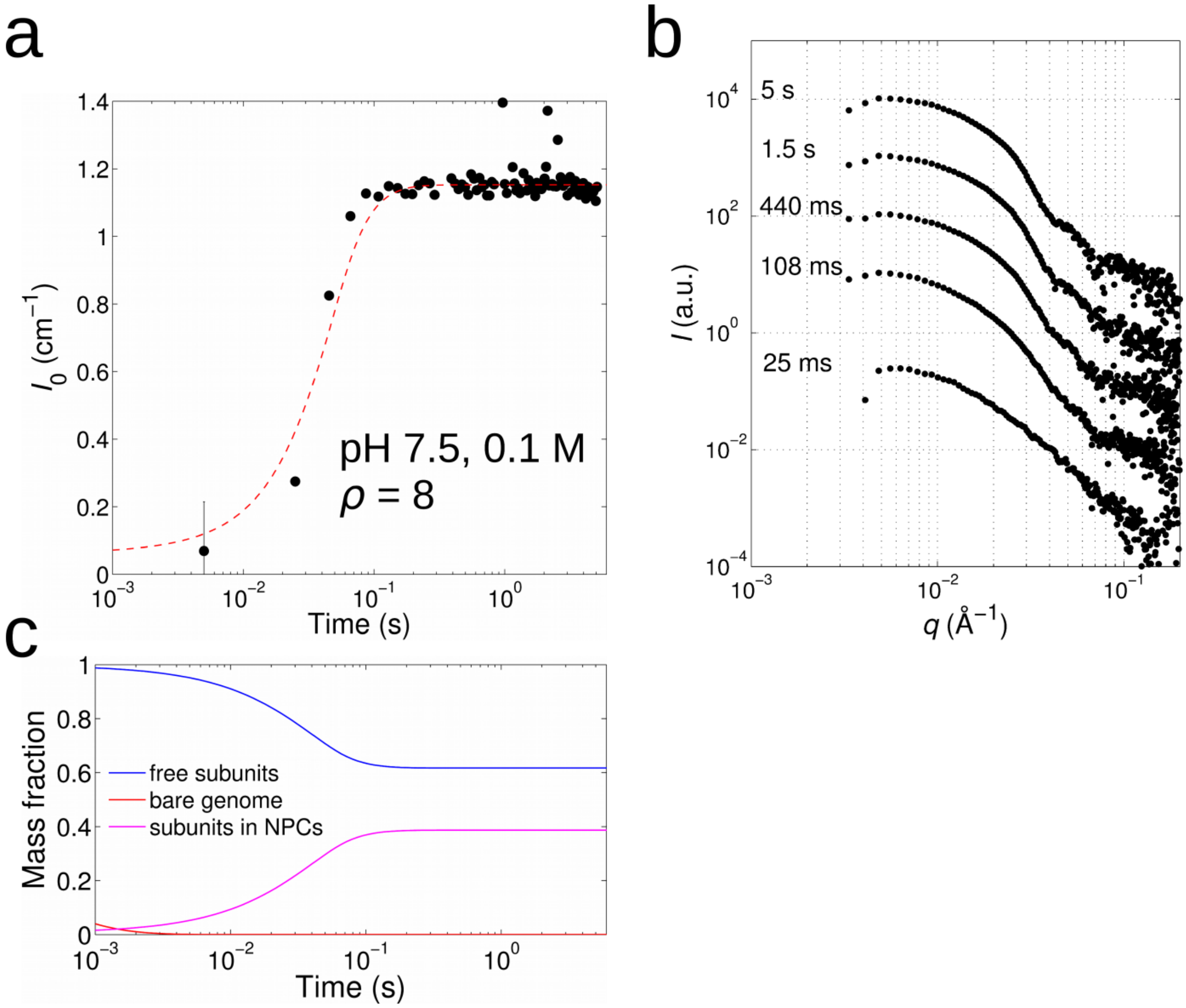


**Supplementary Fig. 6 | Binding kinetics of subunits on genome at $\rho$ = 8. a**, The experimental forward scattering intensity $I_0$ (black discs) is fitted with the scattering intensity (dashed red line) inferred from the templated assembly model described in Supplementary Methods. Subunits and genome were mixed at a mass ratio $\rho$ of 8 in a final buffer solution at pH 7.5 and ionic strength 0.1 M. The fitting forward reaction rate constant $k^+$ is 451 $\mu M^{-1}.s^{-1}$ and the backward reaction rate constant $k^-$ is 6300 $s^{-1}$. Error bars are defined as s.e.m. and were obtained by propagating the standard deviations of photon counts (see Methods). **b**, Scattering patterns at different time points for the same experiment. **c**, Mass fractions inferred from the fit with the templated assembly model versus time: fraction of free subunits $X_S/c_S$ (blue), fraction of bare genome $X_G/c_G$ (red) and fraction of subunits in NPCs $\sum NX_N/c_S$ (magenta).

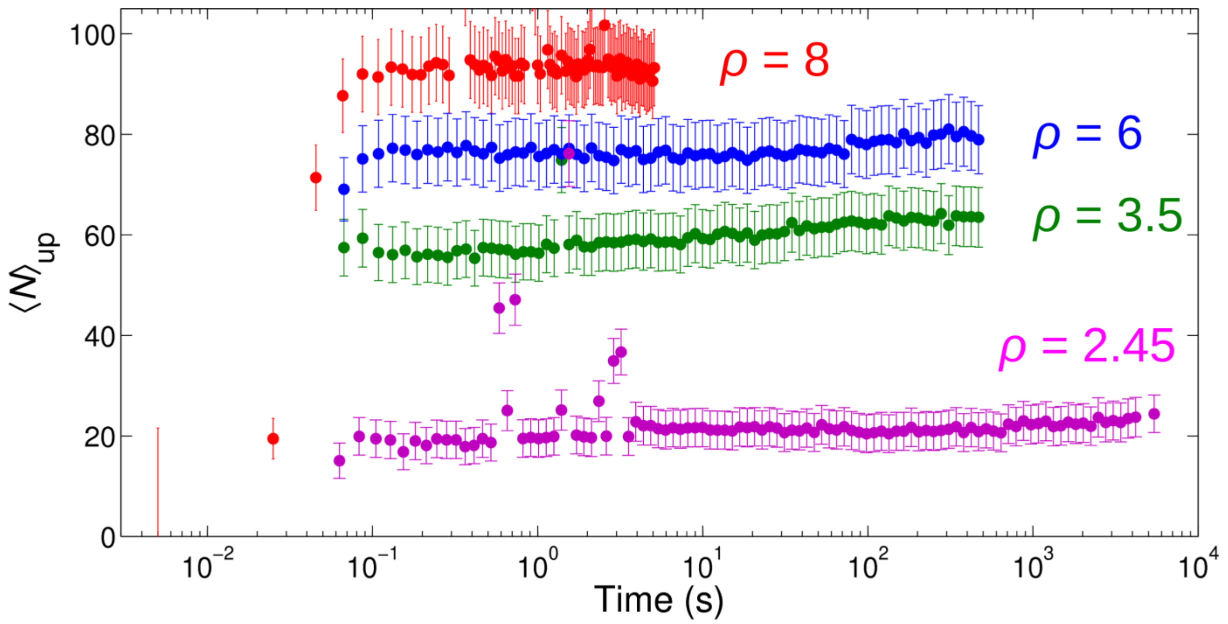


**Supplementary Fig. 7 | Binding kinetics of subunits on genome at various *ρ*.** $\langle N \rangle_{\mathrm{up}}$ as a function of time after mixing subunits and genome with different mass ratios $\rho$ in a buffer solution at pH 7.5 with a final ionic strength of 0.1 M. Error bars are defined as s.e.m. and were obtained by propagating the standard deviations of photon counts (see Methods).

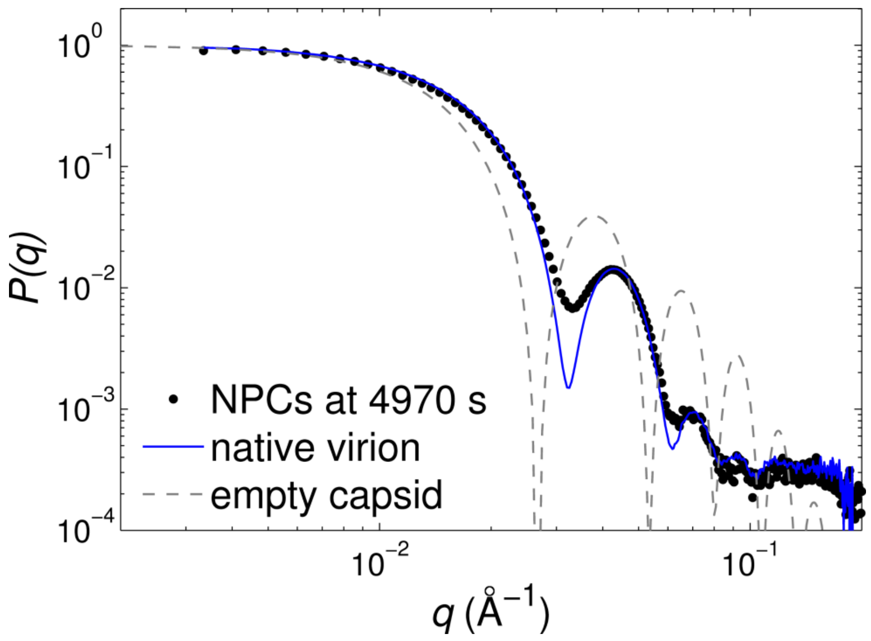


**Supplementary Fig. 8 | Comparison between the form factors $P(q)$ of different species.** The form factor of relaxing NPCs (black discs) was obtained from the last scattering curve ($t$ = 4970 s) of the experiment presented on Fig. 3a in the main text. The scattering intensities were beforehand de-noised by retaining the first five components of a singular value decomposition[2]. The concentration of free subunits $X_S$ was estimated by the procedure described in Supplementary Methods for the determination of $\langle N \rangle_{\mathrm{up}}$. Given that the form factor of the subunit $P_{\mathrm{S}}(q)$ was known (Supplementary Fig. 1), the scattering intensity arising from free subunits, that is, $\Delta b_{\mathrm{S}}^2 X_{\mathrm{S}} P_{\mathrm{S}}(q)$, was subtracted from the last scattering curve to yield the scattering intensity arising solely from NPCs. Their form factor was then computed by normalising their scattering intensity. The form factor of the native virion (blue solid line) was measured from a solution of purified virions in a buffer solution at pH 4.8 and ionic strength 50 mM. The form factor of the empty capsid (gray dashed line) was calculated from the crystal structure of CCMV empty capsid (PDB reference 1ZA7) by the CRYSOL package[3]. This graph shows that the structure of relaxing NPCs was close to that of native virions. It also demonstrates that the contribution of empty capsids coexisting with NPCs was negligible in those conditions.

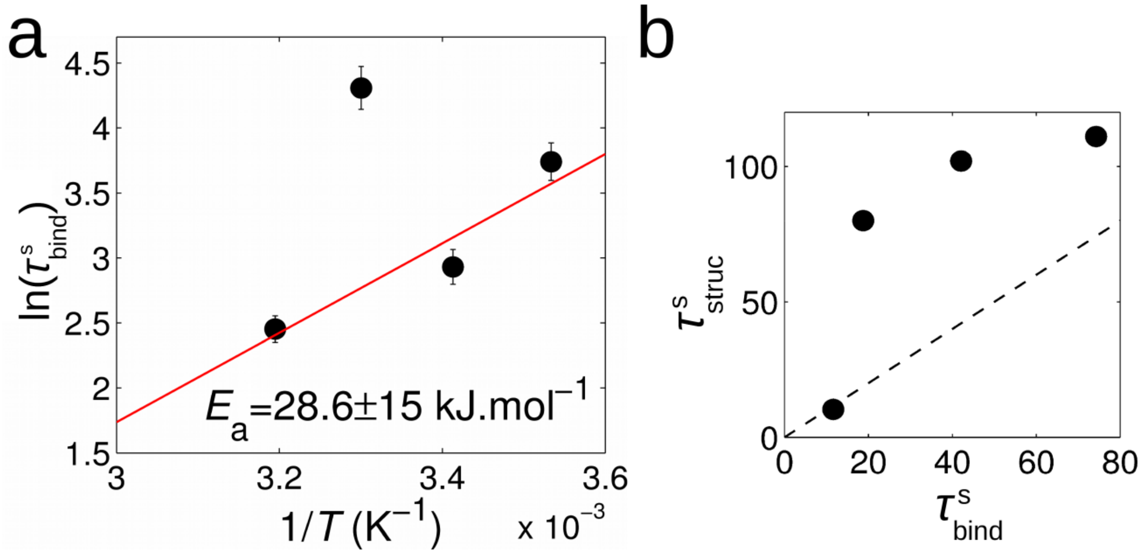


**Supplementary Fig. 9 | Short timescales for the relaxation of NPCs into virions. a**, Logarithm of $\tau^{s}_{bind}$ as a function of the inverse of temperature for experiments performed at 10, 20, 30 and 40°C. $\tau^{s}_{bind}$ is the shortest decay time of the double exponential fits shown on Supplementary Fig. 10. The red line is a linear fit obtained by excluding the data point at 30°C and giving an activation energy $E_a$. Error bars are defined as s.e.m. and were obtained by propagating the standard deviations of photon counts (see Methods). **b**, Shortest structural time $\tau^{s}_{struc}$ versus $\tau^{s}_{bind}$ for experiments performed at 10, 20, 30 and 40°C. $\tau^{s}_{struc}$ is the shortest decay time of the double exponential fits shown on Supplementary Fig. 11.

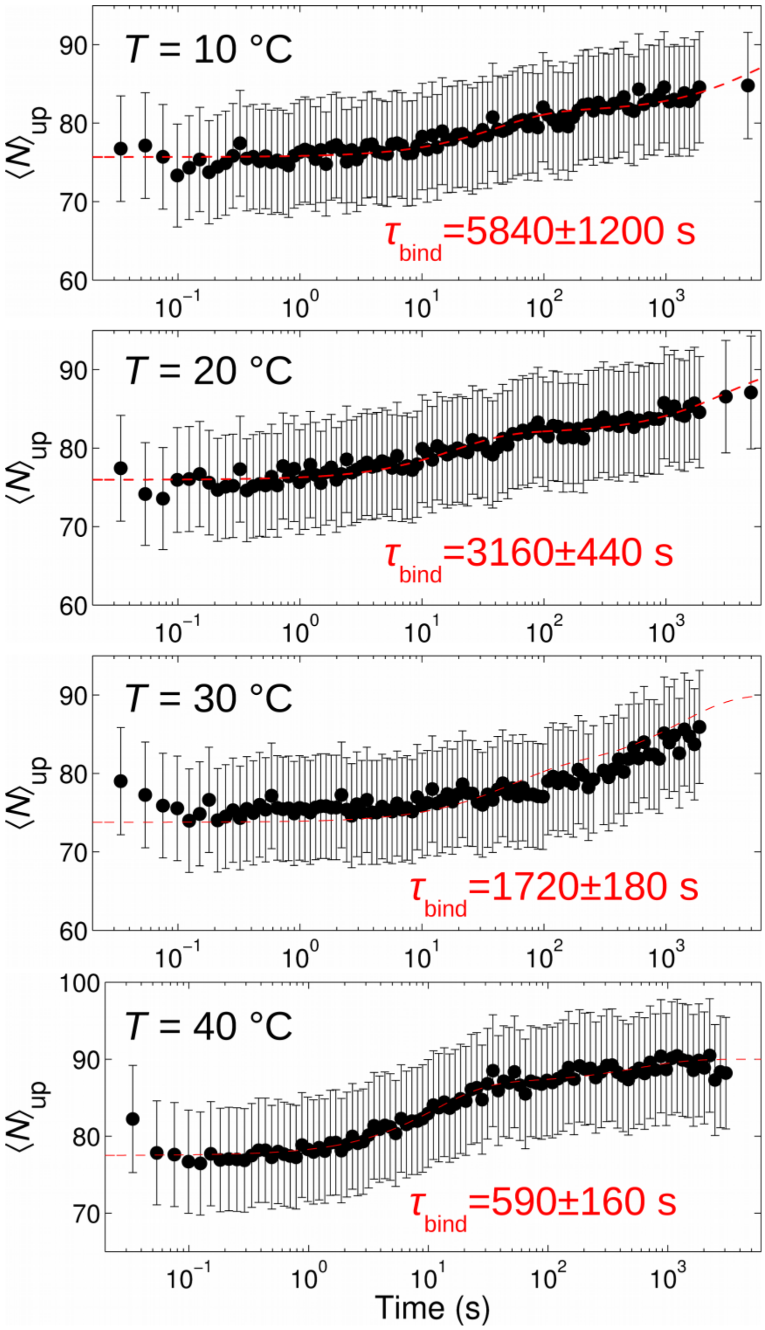


**Supplementary Fig. 10 | Binding time for the relaxation of NPCs into virions at various temperatures.** $\langle N \rangle_{\rm up}$ (black discs) as a function of time at 10, 20, 30 and 40°C. NPCs with a subunit-to-genome mass ratio $\rho = 5.5$, initially at pH 7.5 and an ionic strength of 10 mM, were rapidly mixed with a buffer solution bringing the pH to 5.2 and the ionic strength to 55 mM. The red dashed lines are double exponential decay fits, whose largest decay time was identified as the binding time $\tau_{\rm bind}$. The uncertainty of $\tau_{\rm bind}$ was computed by taking into account the error bars on $\langle N \rangle_{\rm up}$. Error bars are defined as s.e.m. and were obtained by propagating the standard deviations of photon counts (see Methods).

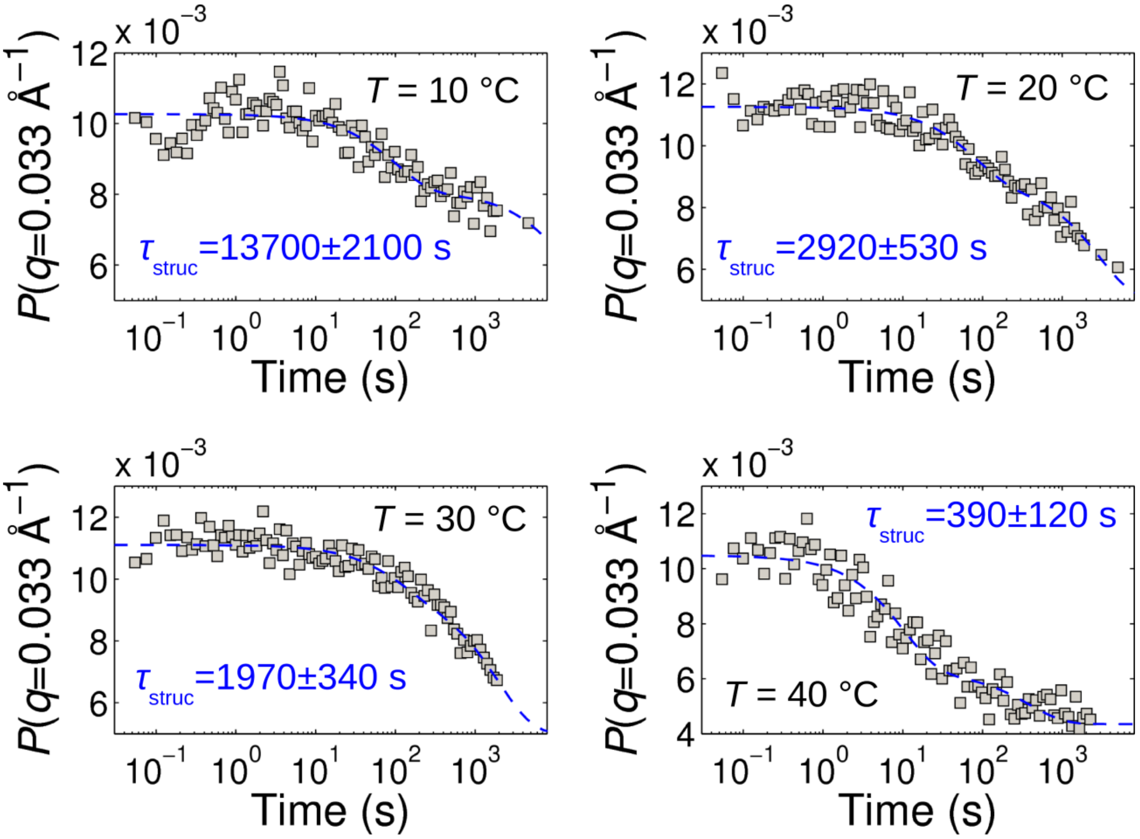


**Supplementary Fig. 11 | Structural time for the relaxation of NPCs into virions at various temperatures.** Value of the form factors of NPCs at $q = 0.033$ Å$^{-1}$ versus time at 10, 20, 30 and 40°C. The form factors of NPCs were computed as described in Supplementary Fig. 8. NPCs with a subunit-to-genome mass ratio $\rho = 5.5$, initially at pH 7.5 and an ionic strength of 10 mM, were rapidly mixed with a buffer solution bringing the pH to 5.2 and the ionic strength to 55 mM. The blue dashed lines are double exponential decay fits, whose largest decay time was identified as the structural relaxation time $\tau_{\mathrm{struc}}$.

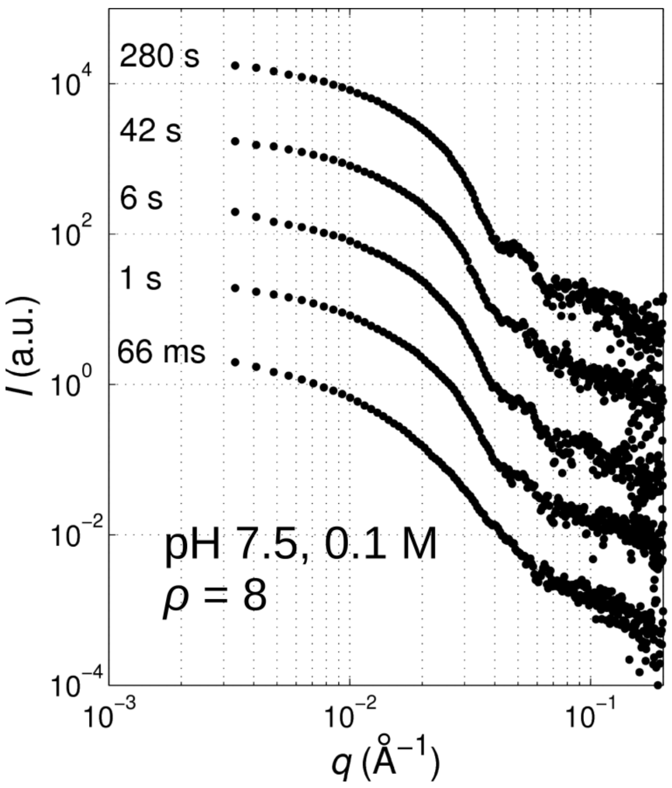


**Supplementary Fig. 12 | Self-assembly kinetics of PSS-filled capsids.** Scattering patterns collected at different time points. Subunits and PSS were mixed at a mass ratio of 8 in a final buffer solution at pH 7.5 and ionic strength 0.1 M.

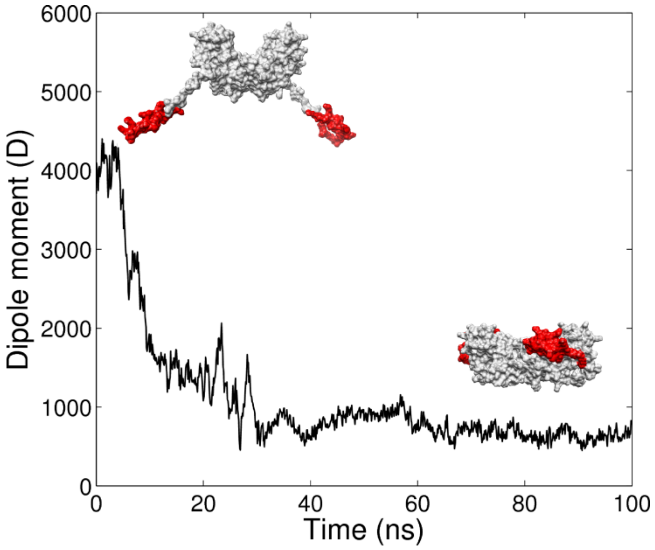


**Supplementary Fig. 13 | Dipole moment of a subunit computed by molecular dynamics simulation.** The simulation was carried out with the GROMACS package[4] by using the AMBER force fields. The crystal structure of a CCMV dimer (PDB reference 1ZA7) was used and the missing N-terminal cationic residues (in red on the molecular structure) were added in a random conformation. The ionisation state of each residue was set in accordance with a pH of 7.5. The solvent was explicit and 0.1 M of monovalent salt ($Na^+$, $Cl^-$) was incorporated in the simulation box. The graph shows that the two N-terminal arms fold against the body of the subunit, which lowers considerably the dipole moment.

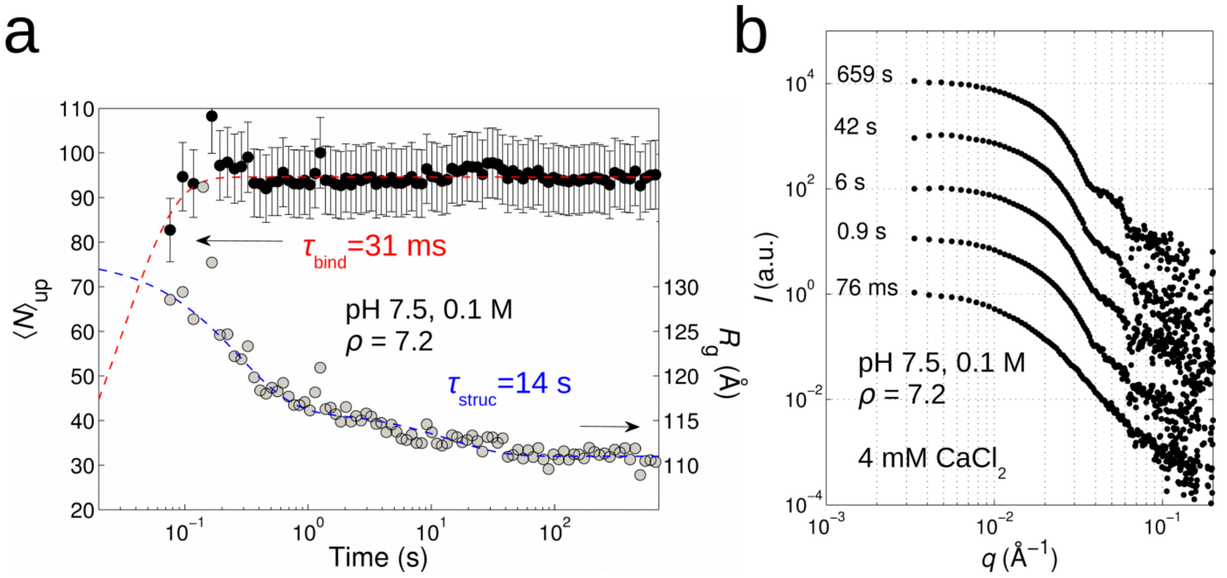


**Supplementary Fig. 14 | Self-assembly dynamics of NPCs in the presence of divalent cations. a**, $\langle N \rangle_{up}$ (black discs) and $R_g$ (light gray discs) as a function of time after mixing subunits and genome at a mass ratio of 7.2 in a final buffer solution at pH 7.5, ionic strength 0.1 M and containing 4 mM $CaCl_2$. The red and blue dashed lines are single and double exponential decay fits returning $\tau_{bind}$ and $\tau_{struc}$ respectively. The red dashed line starts from the origin because in this experiment, $\langle N \rangle_{up}(t = 0) = 0$. Interestingly, $\langle N \rangle_{up}(t \to +\infty)$ amounts to 94, in good agreement with the expected value of 90 determined by the fit of Fig. 2e for $\rho = 7.2$. Error bars are defined as s.e.m. and were obtained by propagating the standard deviations of photon counts (see Methods). **b**, Some scattering patterns collected at different time points for the same experiment.

## Supplementary Methods

**Templated assembly model.** The dynamics of nucleoprotein complexes (NPCs) can be interpreted in terms of a templated assembly model:

$$\mathrm{C}_N + \mathrm{S} \underset{k^-}{\overset{k^+}{\rightleftharpoons}} \mathrm{C}_{N+1} \quad (1)$$

Here, $S$ are free subunits, $\mathrm{C}_N$ are $N$th-order complexes with $N = 0,1,2\ldots,+\infty$ and $\mathrm{C}_0$ stand for bare genome. In principle, the forward and backward reaction rate constants $k^+$ and $k^-$ depend on $N$, but for the sake of simplicity, we will keep them constant. Mass conservation imposes two constraints on the molar concentrations $X_N$, namely

$$\begin{cases} X_\mathrm{G} + \sum_{N=1}^{+\infty} X_N = c_\mathrm{G} \\ X_\mathrm{S} + \sum_{N=1}^{+\infty} N X_N = c_\mathrm{S} \end{cases} \quad (2)$$

where $X_\mathrm{S}$ and $X_\mathrm{G}$ denote the molar concentrations of free subunits and bare genome while $c_\mathrm{S}$ and $c_\mathrm{G}$ are the total molar concentrations of subunits and genome in the solution. The evolution of the molar concentrations is given by a set of ordinary differential equations describing the reactions of Supplementary Equation 1:

$$\begin{cases} \dfrac{dX_\mathrm{G}}{dt} & = & k^- X_1 - k^+ X_\mathrm{S} X_\mathrm{G} \\ & \vdots & \\ \dfrac{dX_N}{dt} & = & k^-(X_{N+1} - X_N) + k^+(X_{N-1} - X_N) X_\mathrm{S} \\ & \vdots & \end{cases} \quad (3)$$

At equilibrium, Supplementary Equation 3 leads to $X_N = (K X_\mathrm{S})^N X_\mathrm{G}$ with $K = k^+/k^-$ the equilibrium constant. Supplementary Equation 2 then gives a quadratic equation in $X_\mathrm{S}$:

$$K X_\mathrm{S}^2 - [1 + K(c_\mathrm{S} + c_\mathrm{G})] X_\mathrm{S} + c_\mathrm{S} = 0 \quad (4)$$

and, assuming $c_\mathrm{S} \gg c_\mathrm{G}$, we arrive at a classical result in the theory of self-assembly, namely,

$$X_S \approx \begin{cases} c_\mathrm{S}, & c_\mathrm{S} < K^{-1} \\ K^{-1}, & c_\mathrm{S} \geq K^{-1} \end{cases} \quad (5)$$

where $K^{-1}$ turns out to be a critical subunit concentration above which NPCs are formed (Supplementary Fig. 4). At any time step, the mean number of subunits per NPC is given by

$$\langle N\rangle = \frac{\sum_{N=0}^{+\infty} N X_N}{X_\mathrm{G} + \sum_1^{+\infty} X_N} = \frac{c_\mathrm{S} - X_\mathrm{S}}{c_\mathrm{G}} \tag{6}$$

and since in our experiments $c_\mathrm{S}$ was larger than $K^{-1}$, we obtain at equilibrium

$$\langle N\rangle(t \to +\infty) \approx \frac{c_\mathrm{S} - K^{-1}}{c_\mathrm{G}} \tag{7}$$

The critical concentration can also be written as $K^{-1} = N_\mathrm{A}^{-1} v_0^{-1} \exp(G_\mathrm{bind}/k_\mathrm{B}T)$, with $N_\mathrm{A}$ the Avogadro constant, $v_0$ a standard state volume (taken as $4/3\,\pi R_\mathrm{g}^3$ with $R_\mathrm{g} = 33$ Å the radius of gyration of subunits), $G_\mathrm{bind}$ the binding free energy of one subunit to one NPC, $k_\mathrm{B}$ the Boltzmann constant and $T$ the temperature.

The templated assembly model can be further refined by introducing the maximal number of subunits per NPC, $N_\mathrm{max}$. The mass conservation (Supplementary Equation 2) becomes

$$\begin{cases} X_\mathrm{G} + \sum_{N=1}^{N_\mathrm{max}} X_N = c_\mathrm{G} \\ X_\mathrm{S} + \sum_{N=1}^{N_\mathrm{max}} N X_N = c_\mathrm{S} \end{cases} \tag{8}$$

which gives a complex equation relating $X_\mathrm{S}$ to $c_\mathrm{S}$:

$$X_\mathrm{S} + c_\mathrm{G} \frac{K X_\mathrm{S}}{1 - K X_\mathrm{S}} \frac{1 - (N_\mathrm{max}+1)(K X_\mathrm{S})^{N_\mathrm{max}} + N_\mathrm{max}(K X_\mathrm{S})^{N_\mathrm{max}+1}}{1 - (K X_\mathrm{S})^{N_\mathrm{max}+1}} = c_\mathrm{S} \tag{9}$$

Since $N_\mathrm{max} \gg 1$, Supplementary Equation 4 is a good approximate solution for $K X_\mathrm{S} < 1$ and describes well the critical concentration. For $K X_\mathrm{S} > 1$, we can find another approximate solution that describes the saturation of the genome by the bound subunits:

$$X_\mathrm{S} + \frac{c_\mathrm{G}}{1 - K X_\mathrm{S}} = c_\mathrm{S} - c_\mathrm{G} N_\mathrm{max} \tag{10}$$

In all cases, assuming $c_\mathrm{S} \gg c_\mathrm{G}$, the mean number of subunits per NPC at the equilibrium given by Supplementary Equations 9 and 6 can be conveniently approximated by a piecewise function (see Supplementary Fig. 5):

$$\langle N\rangle(t\to+\infty) \approx \begin{cases} 0, & c_\mathrm{S} \le K^{-1} \\ \dfrac{c_\mathrm{S}-K^{-1}}{c_\mathrm{G}}, & K^{-1} \le c_\mathrm{S} \le K^{-1}+c_\mathrm{G}N_\mathrm{max} \\ N_\mathrm{max}, & c_\mathrm{S} \ge K^{-1}+c_\mathrm{G}N_\mathrm{max} \end{cases} \quad (11)$$

**Determination of $\langle N\rangle_\mathrm{up}$ and $\langle N\rangle(t\to+\infty)$.** The forward scattering intensity $I_0$ of a solution containing NPCs is given by[5]

$$I_0 = \Delta b_\mathrm{S}^2 X_\mathrm{S} + \sum_{N=0}^{+\infty} X_N(\Delta b_\mathrm{G} + \Delta b_\mathrm{S} N)^2 \quad (12)$$

where $\Delta b_\mathrm{S}$ and $\Delta b_\mathrm{G}$ are the excess scattering lengths of subunit and genome respectively ($\Delta b_\mathrm{G} \gg \Delta b_\mathrm{S} > 0$), $X_\mathrm{S}$ the molar concentration of free subunits, and $X_N$ the molar concentration of the $N$th-order complexes. We denote here $X_0 \equiv X_\mathrm{G}$ the molar concentration of bare genome. By using the mass conservation (Supplementary Equation 2), the mean number of subunits per NPC reads

$$\langle N\rangle = \frac{\sum_{N=0}^{+\infty} N X_N}{X_\mathrm{G} + \sum_{N=1}^{+\infty} X_N} = \frac{\sum_{N=0}^{+\infty} N X_N}{c_\mathrm{G}} \quad (13)$$

with $c_\mathrm{G}$ the total molar concentration of genome. A similar expression can be obtained for $\langle N^2\rangle$. Supplementary Equation 12 can then be developed and reorganised into

$$\Delta b_\mathrm{S}^2 c_\mathrm{G}\langle N^2\rangle + (2\Delta b_\mathrm{G}\Delta b_\mathrm{S} - \Delta b_\mathrm{S}^2) c_\mathrm{G}\langle N\rangle - (I_0 - I_0^*) = 0 \quad (14)$$

where we have used the mass conservation (Supplementary Equation 2) on the total molar concentration of subunits, i.e., $c_\mathrm{S} = X_\mathrm{S} + \sum_{N=0}^{+\infty} N X_N$, and with $I_0^* \equiv \Delta b_\mathrm{G}^2 c_\mathrm{G} + \Delta b_\mathrm{S}^2 c_\mathrm{S}$ the scattering intensity produced by the mixture of subunits and genome prior to complexation. $\langle N^2\rangle$ can be rewritten $\langle N\rangle^2 + \sigma_N^2$ with $\sigma_N$ the standard deviation of the number of subunits per NPC. $\langle N\rangle$ can be computed by solving a quadratic equation and is then a function of the unknown parameter $\sigma_N$:

$$\langle N \rangle = \sqrt{\Gamma^2 + \left(\frac{I_0 - I_0^*}{\Delta b_S^2 c_G} - \sigma_N^2\right)} - \Gamma \tag{15}$$

with $\Gamma = \Delta b_G/\Delta b_S - 1/2 \approx \Delta b_G/\Delta b_S > 0$. It is straightforward to see that an upper limit for $\langle N \rangle$ is given by its value at $\sigma_N = 0$, i.e., $\langle N \rangle \leq \langle N \rangle(\sigma_N = 0) \equiv \langle N \rangle_{\mathrm{up}}$.

At the equilibrium, we can inject the analytical expressions given by the templated assembly model into Supplementary Equation 14. We then arrive at a quadratic equation relating the mean number of subunits per NPC to the forward scattering intensity, both evaluated at the equilibrium:

$$\langle N \rangle(t \to +\infty) = \sqrt{\frac{1}{4}\Gamma^2 + \frac{I_0(t \to +\infty) - I_0^*}{2\Delta b_S^2 c_G}} - \frac{1}{2}\Gamma \tag{16}$$

For TR-SAXS experiments, the excess scattering lengths were estimated from measurements on subunits, genome and purified virions. We found $\Delta b_G = 0.9929$ cm$^{-0.5}$.µM$^{-0.5}$ and $\Delta b_S = 0.0201$ cm$^{-0.5}$.µM$^{-0.5}$. In the case of PSS, the excess scattering length was calculated from the electron density of PSS and we arrived at $\Delta b_{PSS} = 0.928$ cm$^{-0.5}$.µM$^{-0.5}$. For SANS experiments in 68% $D_2O$, $\Delta b_G$ vanished since the solvent contrast matched the genome, and $\Delta b_S$ was estimated to be 0.0141 cm$^{-0.5}$.µM$^{-0.5}$. The uncertainties on $\langle N \rangle_{\mathrm{up}}$ included the experimental uncertainties on scattering intensities and concentrations.

## Supplementary References


1. Svergun, D.I. Determination of the regularization parameter in indirect-transform methods using perceptual criteria. *J. Appl. Crystallogr.* **25**, 495-503 (1992).
2. Henry, E.R. & Hofrichter, J. Singular value decomposition - Application to analysis of experimental data. *Methods Enzymol.* **210**, 129-192 (1992).
3. Svergun, D., Barberato, C. & Koch, M.H.J. CRYSOL - A program to evaluate x-ray solution scattering of biological macromolecules from atomic coordinates. *J. Appl. Crystallogr.* **28**, 768-773 (1995).
4. Berendsen, H.J.C., Vanderspoel, D. & Vandrunen, R. GROMACS: A message-passing parallel molecular-dynamics implementation. *Comput. Phys. Commun.* **91**, 43-56 (1995).
5. Svergun, D.I. & Koch, M.H.J. Small-angle scattering studies of biological macromolecules in solution. *Rep. Prog. Phys.* **66**, 1735-1782 (2003).
